# Significant differences when using creatinine, modification of diet in renal disease, or cystatin C for estimating glomerular filtration rate in ICU patients

**DOI:** 10.3109/03009734.2010.526724

**Published:** 2011-02-11

**Authors:** Miklós Lipcsey, Mia Furebring, Sten Rubertsson, Anders Larsson

**Affiliations:** ^1^Section of Anaesthesiology & Critical Care, Department of Surgical Sciences, Uppsala University Hospital, UppsalaSweden; ^2^Section of Infectious Diseases, Department of Medical Sciences, Uppsala University Hospital, UppsalaSweden; ^3^Section of Clinical Chemistry, Department of Medical Sciences, Uppsala University Hospital, UppsalaSweden

**Keywords:** Cystatin C, glomerular filtration rate, human, intensive care, kidney, MDRD

## Abstract

**Background:**

Renal dysfunction is associated with increased morbidity and mortality in intensive care patients. In most cases the glomerular filtration rate (GFR) is estimated based on serum creatinine and the Modification of Diet in Renal Disease (MDRD) formula, but cystatin C-estimated GFR is being used increasingly. The aim of this study was to compare creatinine and MDRD and cystatin C-estimated GFR in intensive care patients.

**Methods:**

Retrospective observational study was performed, on patients treated within the general intensive care unit (ICU) during 2004–2006, in a Swedish university hospital.

**Results:**

GFR markers are frequently ordered in the ICU; 92% of the patient test results had cystatin C-estimated GFR (eGFR_cystatinC_) ≤ 80 mL/min/1.73 m^2^, 75% had eGFR ≤ 50 mL/min/1.73 m^2^, and 30% had eGFR ≤ 20 mL/min/1.73 m^2^. In contrast, only 46% of the patients had reduced renal function assessed by plasma creatinine alone, and only 47% had eGFR_MDRD_ ≤ 80 mL/min/1.73 m^2^. The mean difference between eGFR_MDRD_ and eGFR_cystatinC_ was 39 mL/min/1.73 m^2^ for eGFR_cystatinC_ values ≤ 60 mL/min/1.73 m^2^.

**Conclusions:**

GFR is commonly assessed in the ICU. Cystatin C-estimated GFR yields markedly lower GFR results than plasma creatinine and eGFR_MDRD_. Many pharmaceuticals are eliminated by the kidney, and their dosage is adjusted for kidney function. Thus, the differences in GFR estimates by the methods used indicate that the GFR method used in the intensive care unit may influence the treatment.

## Introduction

Glomerular filtration rate (GFR) is generally accepted as the best overall indicator of renal function and is therefore an important marker for renal disease. Reduced GFR is one of the most important complications in critically ill patients and is associated with increased morbidity and mortality in the intensive care unit (ICU) population (1–4). Furthermore, reduced GFR influences the clearance of many pharmaceuticals used today. Thus, in many cases the recommended dose of a drug has to be adjusted depending on the patient's GFR. For instance, the dosage of antibiotics and cytotoxic drugs is usually prescribed according to GFR. Monitoring renal function is thus very important in the management of intensive care patients, and GFR markers are frequently used for this purpose, necessitating convenient and reliable GFR markers. Inulin, iohexol, and ^51^Cr-EDTA clearances are considered as ‘gold standards’ for GFR measurements in Sweden (5–7). The disadvantage with these assays, and creatinine clearance with urine collection, is that they are cumbersome, costly, and associated with long turn-around times, which may delay initiation and adjustment of treatment. They are thus less suitable for an intensive care unit requiring rapid decisions and actions. Thus, endogenous markers are usually preferred in the intensive care setting. The ideal endogenous marker should have a stable production rate, be unaffected by pathological changes, lack protein binding, be freely filtered through the glomeruli, and lack reabsorption or secretion. To date, no such marker has been identified.

Serum or plasma creatinine is the most commonly used GFR marker (8,9). Creatinine is an inexpensive test widely available in clinical chemistry laboratories, but the assay outcome is hampered by the influence of several extrarenal factors such as age, gender, muscle mass, physical activity, and diet (10,11). It is also insensitive for small decreases in GFR, in the so-called creatinine-blind GFR area, due to the non-linear relationship between plasma concentration and GFR (12).

Cystatin C is an endogenous polypeptide that is more sensitive than serum creatinine for the detection of small decreases in GFR and is reported not to be influenced by inflammation, liver function, age, gender, muscle mass, physical activity, or diet (13). Human cystatin C has a plasma clearance of cystattin C of 94% of the generally used GFR-marker, ^51^Cr-EDTA in the rat (14).

Previously, cystatin C assays were hampered by the limited availability of the test, but cystatin C methods have now been developed for clinical chemistry laboratories making the test widely accessible. Cystatin C can thus be analyzed with short turn-round times providing rapid test results for intensive care, at costs comparable to plasma creatinine analysis.

A recent meta-analysis has shown that cystatin C is superior to plasma creatinine as a marker of renal function (15). Another study has suggested that cystatin C is superior to creatinine as a GFR marker in critically ill patients (16).

The aim of this study was to assess the prevalence of reduced GFR in this patient group using creatinine and Modification of Diet in Renal Disease (MDRD) and cystatin C-estimated GFR, as several pharmaceuticals are prescribed according to renal function, and to investigate the request frequency of laboratory markers in an ICU, with special reference to GFR markers.

## Materials and methods

### Study population

This study included all patients treated within the general intensive care unit, Uppsala University Hospital, during the time period 1 January 2004 to 1 September 2006. The total number of tests per year, presented in Table I, was based on all requests during the same time period. The Table thus also includes requests for patients below 16 years of age and requests without a valid test result (e.g. samples that could not be analyzed due to insufficient sample volumes). For comparison between cystatin C and creatinine results, only valid test results from patients older than 16 years of age were included. Blood samples for cystatin C analyses were usually collected early in the morning and not more than once a day.

**Table I.** GFR markers, drug levels in plasma among the most frequently ordered tests during the study period 2004–2006 (mean number of tests per year).

Test	*n*
1. Blood gases (ABL 725)	50725
2. Creatinine	3089
3. Blood cell counts	3070
4. C-reactive protein	3063
5. Activated partial thromboplastin time	2883
6. Prothrombin complex	2797
7. Bilirubin, direct method	1972
8. Alanine aminotransferase (ALT)	1972
17. Pt-GFR (CystC-calculated)	875
25. Vancomycin	182
28. Tobramycin	120
31. Digoxin	84
36. Gentamycin	56

Pt-GFR, Patient-Glomerular filtration rate.

### Intensive care unit (ICU)

The study was conducted in a ten-bed general intensive care unit admitting patients from medical and surgical specialties in a university hospital. Approximately 1,200–1,500 patients are treated in this unit each year, with a mean admittance time of 2.5 days. The unit has an ABL 725 (Radiometer, Copenhagen, Denmark) blood gas instrument for point of care testing in the ward.

### Sample collection

The samples for creatinine and cystatin C analyses were collected in gel tubes with lithium-heparin (LH PST^™^ II, BD Vacutainer Systems, Plymouth, UK).

### Plasma cystatin C and cystatin C-estimated GFR (eGFR)

Plasma cystatin C measurements were performed by latex-enhanced reagent (N Latex cystatin C, Dade Behring, Deerfield, IL, USA) using a Behring BN ProSpec analyzer (Dade Behring). The total analytical imprecision of the method was 4.8% at 0.56 mg/L and 3.7% at 2.85 mg/L. The cystatin C results are reported as a cystatin C-estimated GFR (eGFR) in mL/min/1.73 m^2^ (17). GFR in mL/min/1.73 m^2^ was calculated from cystatin C results in mg/mL by the equation *y* = 77.24 × (Cystatin C result)^-1.2623^ (17). The reference value for cystatin C-estimated GFR (eGFR) was ≥ 80 mL/min/1.73 m^2^.

### Plasma creatinine and Modification of Diet in Renal Disease (MDRD) formula-estimated GFR (eGFR)

Plasma creatinine was analyzed with a modified kinetic Jaffé reaction on an Architect Ci8200 analyzer (Abbott, Abbott Park, IL, USA) and reported as SI units (μmol/L). The method is isotope dilution mass spectrometry (IDMS)-calibrated in collaboration with the Swedish external quality assurance organization (Equalis, Uppsala, Sweden). The total analytical imprecision of the creatinine method was 4.8% at both 94 and 337 μmol/L. The reference interval for creatinine concentration in adult males was 60–100 μmol/L. The reference interval for creatinine concentration in adult females was 50–90 μmol/L. eGFR_MDRD_ was calculated from creatinine using the MDRD formula: eGFR = 175 × (creatinine (μmol/L)/88.4)^−1.154^ × age (years)^−0.203^ × 0.742 (if female) (18). The factor for African Americans was not used.

### Statistical calculations

All calculations were performed with the statistical software package Statistica 7.0 (StatSoft, Tulsa, OK, USA). Associations between continuous variables were tested with Spearman's rank correlation analysis (*R*). *P* values < 0.05 were regarded as statistically significant throughout the study.

## Results

### Most frequently ordered tests

The most frequently ordered test was arterial blood gas analysis. Creatinine was the second most frequently ordered test on the list (Table I). Aminoglycosides, vancomycin, and digoxin were the most frequently ordered drug tests.

### Cystatin C and creatinine plasma levels

During the studied time period there were 1,838 cystatin C test results. Of these requests 1,151 (63%) were for male patients and 687 (37%) were for female patients. The mean age of the patients was 62 years (62 for both males and females).

Median cystatin C concentration in the cohort was 1.96 mg/L (interquartile range 1.38–2.81 mg/L), and mean value was 2.23 mg/L. For males the median cystatin C concentration was 1.99 mg/L (interquartile range 1.39–2.81 mg/L), and for females the median cystatin C concentration was 1.90 mg/L (interquartile range 1.36–2.83 mg/L).

During the time period there were 7,566 requests for creatinine. Of these requests 4,573 (60%) were for male patients, and 2,993 (40%) were for female patients.

Median creatinine concentration in the cohort was 91 μmol/L (interquartile range 67–153 μmol/L), and mean value was 131 μmol/L. For males the median creatinine concentration was 99 μmol/L (interquartile range 73–166 μmol/L), and for females the median creatinine concentration was 80 μmol/L (interquartile range 60–132 μmol/L). A total of 2,247 out of 4,573 test results for males were above the reference interval (49%); 1,234 out of 2,993 test results for females were above the reference interval (41%).

In 88% of the patients, plasma cystatin C values indicated reduced kidney function, whereas only 46% of the patients had reduced kidney function as evaluated by their plasma creatinine levels.

### eGFR calculated from cystatin C values

Median eGFR_cystatinC_ in the study group was 30.3 mL/min/1.73 m^2^ (interquartile range 17.9–50.1 mL/min/1.73 m^2^), and mean value was 37.6 mL/min/1.73 m^2^. For males the median eGFR was 29.7 mL/min/1.73 m^2^ (interquartile range 18.1–49.7 mL/min/1.73 m^2^), and for females the median eGFR_cystatinC_ was 31.7 mL/min/1.73 m^2^ (interquartile range 17.9–51.4 mL/min/1.73 m^2^).

Out of the 1,838 eGFR_cystatinC_ results, only 146 results (7.9%) were within the reference range, while 92.1% of the results were below the reference interval. Out of the 1,838 eGFR results, 454 results (25%) were higher than 50 mL/min/1.73 m^2^, and 1,279 (70%) were higher than 20 mL/min/1.73 m^2^.

There was a strong negative correlation between age and eGFR (*R* = −0.316, *P* < 0.0001).

**Figure 1.**
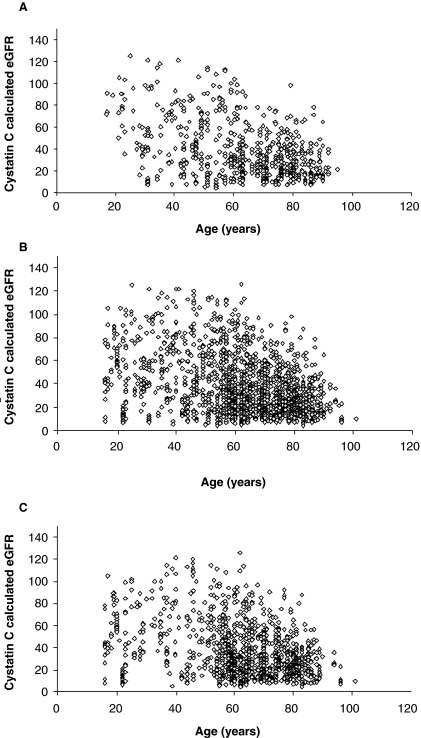
A: Age versus cystatin C-estimated glomerular filtration rate (eGFR) for all patients (*n* = 1,838). B: Age versus cystatin C-estimated glomerular filtration rate (eGFR) for males (*n* = 1,151). C: Age versus cystatin C-estimated glomerular filtration rate (eGFR) for females (*n* = 687).

**Figure 2.**
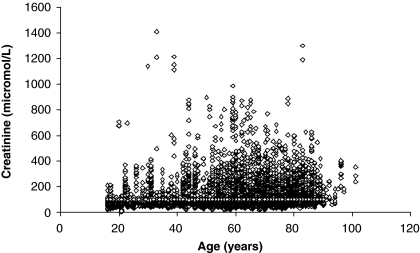
Age versus plasma creatinine for all patients (*n* = 7,566).

Both males (*R* = −0.302; *P* < 0.0001) and females (*R* = −0.344; *P* < 0.0001) showed similar negative correlations (Figures 1 and 2).

### Paired plasma cystatin C and creatinine analyses

There were 1,777 test requests that contained both cystatin C and creatinine results performed on the same test tube. Of these requests 1,110 were for males and 667 for females. These samples were from 734 different patients (median 2.42 samples per patient).

Median creatinine concentration in this subgroup was 127 μmol/L (interquartile range 87–207 μmol/L), and mean value was 167 μmol/L.

For males the median creatinine concentration was 134 μmol/L (interquartile range 95–212 μmol/L), and for females the median creatinine concentration was 111 μmol/L (interquartile range 76–199 μmol/L). A total of 789 out of 1,110 test results for males were in the reference interval (71%); 418 out of 667 test results for females were in the reference interval (63%). In total 1,207 out of 1,777 plasma creatinine results were pathological (68%).

**Figure 3.**
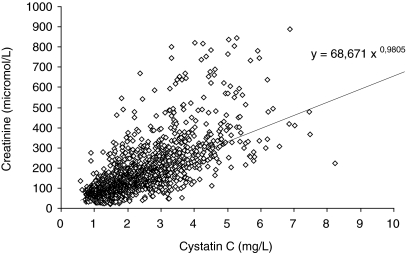
Correlation between cystatin C and creatinine in individual patients (*n* = 1,668).

**Figure 4.**
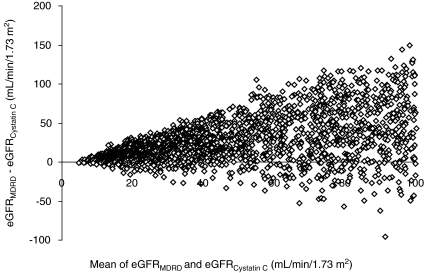
Bland–Altman bias plot for eGFR_cystatinC_ and eGFR_MDRD_. The results are presented as the mean of the two methods (*x*-axis) plotted against the difference between the two methods (*y*-axis) (*n* = 1,777).

Out of the MDRD-estimated GFR results 47% were ≤80 mL/min/1.73 m^2^, 30% were ≤ 50 mL/min/1.73 m^2^, and 7% ≤20 mL/min/1.73 m^2^. Median MDRD eGFR was 48 mL/min/1.73 m^2^ (Table II). Out of the 1,777 cystatin C-estimated GFR results 93% were ≤ 80 mL/min/1.73 m^2^, 74% were ≤50 mL/min/1.73 m^2^, and 23% were ≤20 mL/min/1.73 m^2^. Median MDRD eGFR was 39 mL/min/1.73 m^2^. The Spearman rank correlation between the two methods was *R* = 0.753 (Figure 3). MDRD-estimated GFR yielded higher values than cystatin C-estimated GFR (Figure 4). The mean difference between eGFR_MDRD_ and eGFR_cystatinC_ was 39 mL mL/min/1.73 m^2^ for eGFR_cystatinC_ values ≤ 60 mL/min/1.73 m^2^.

**Table II.** Percentage of patients with GFR below 20, 50, and 80 mL/min/1.73 m^2^, respectively, based on eGFR_MDR__D_ and eGFR_cystatinC_ calculations.

	≤ 20 mL/min/1.73m^2^	≤ 50 mL/min/1.73 m^2^	≤ 80 mL/min/1.73 m^2^
eGFR_cystatinC_	30%	75%	92%
eGFR_MDRD_	7%	30%	47%

## Discussion

Plasma creatinine's role as GFR marker assumes a steady state condition of creatinine distribution. However creatinine levels in plasma and urine are subject to variations during critical illness for several reasons. Critically ill patients often have increased body water volume and decreased muscle mass and may have impaired liver function. Other factors that may influence creatinine production are trauma, fever, and immobilization. There is also the problem of tubular secretion of creatinine to some extent while low urine production may cause tubular reabsorption. Thus, creatinine is not an optimal marker for detection of acute renal failure in intensive care patients; there is on-going search for better GFR markers, and cystatin C and proANP (Atrial natriuretic peptide) have been suggested as alternatives (19). One study that compared cystatin C and creatinine reported that cystatin C and creatinine performed equally well in intensive care patients (20). Two other studies showed cystatin C to be superior to creatinine. One of the studies reported that serum cystatin C detected acute renal failure 1 to 2 days earlier than creatinine (21). The other study compared cystatin C and creatinine with 24-h urine collection and creatinine clearance. Cystatin C was significantly better than creatinine to detect reduced GFR (22). Neither of these studies compared GFR estimated from cystatin C and creatinine with GFR measurement using an exogenous marker such as iohexol, iothalamate, or chromium-51-EDTA.

In this study we show that, apart from arterial blood gas analysis, GFR markers are the most frequently requested tests in the ICU. Furthermore we show that there are considerable differences between the studied markers creatinine, eGFR_MDRD_, and eGFR_cystatinC_ for estimating GFR in ICU patients. Reduced GFR is very common in this ICU population, especially when using cystatin C for estimating GFR. There was a strong negative correlation between age and eGFR_cystatinC_, while the correlation between age and creatinine was less pronounced. This could be expected as both GFR and muscle mass decrease with age. An increased plasma creatinine due to reduced GFR is thus partly disguised by the reduced muscle mass. Cystatin C detected twice as high a frequency of reduced GFR in spite of a high degree of correlation between these two methods.

Plasma creatinine, eGFR_MDRD_, and cystatin C assays can all provide rapid test results. Creatinine often over-estimates GFR in patients with slight reductions of GFR (11). It is also difficult to evaluate creatinine in elderly patients with low muscle mass. These patients may have creatinine values in the normal range due to the combination of low muscle mass and reduced GFR. This is in agreement with the present results with 92% of the cystatin C values indicating reduced kidney function, while only 46% of the creatinine test results indicated a reduced kidney function. A considerable gap between the results, 93% versus 47% for eGFR with cystatin C and eGFR with MDRD, respectively, persisted even when the two methods were performed on the same plasma sample. In this study we used the reference value ≥80 mL/min/1.73 m^2^ regardless of patient age as a decreased eGFR in the range of 50–80 mL/min/1.73 m^2^ was associated with increased mortality in elderly individuals (23–26).

We included plasma creatinine in this comparison as many GFR evaluations in Sweden are based on the creatinine concentration. The GFR estimations from plasma creatinine values are performed in the wards either by a rough estimate based on the creatinine value or by using nomogram for the Cockcroft-Gault equation (8). The equations are very rarely performed utilizing computers.

The problems associated with calculating GFR in the wards have led to the development of formulas to automatically convert cystatin C in mg/L to a calculated GFR in mL/min/1.73 m^2^ (17,27). This is in agreement with guidelines that laboratories should calculate and report an estimated glomerular filtration rate (eGFR) using the MDRD formula with every request for plasma creatinine concentration (28). In the ICU, MDRD and plasma creatinine concentration detected similar numbers of patients with reduced GFR, and both methods detected a lower number of patients with reduced GFR in comparison with cystatin C-estimated GFR.

Cystatin C has also been suggested to be superior to creatinine measurements for intensive care use although this has also been questioned (16,29).

In this study we compared cystatin C as GFR marker with creatinine concentration and eGFR_MDRD_. The study shows that a higher proportion of the intensive care patients had impaired kidney function when using eGFR_cystatinC_. Several of the drugs used in the intensive care unit are eliminated by the kidneys, and their turn-over is thus influenced by the GFR. Determination of plasma concentrations of digoxin, gentamycin, tobramycin, and vancomycin are all among the top 40 test requests in the intensive care unit, and they are all influenced by the GFR. Even though concentration of these drugs can be measured, initial therapy is started based on GFR estimates. Furthermore, levels of several widely used pharmaceuticals with renal elimination are not routinely assessed. Examples of such drugs used in critical care are H_2_-antagonists, beta-blockers, and antibiotics such as penicillins and cephalosporins. Inadequate dosage of these pharmaceuticals may lead to insufficient therapy or adverse effects, which highlight the need for optimal GFR markers or the advantage of pharmaceuticals without renal elimination.

## Conclusions

The present study shows that GFR is frequently assessed in the ICU, and many intensive care patients have reduced GFR, and the study emphasizes the need to monitor GFR in this patient group. The use of cystatin C instead of creatinine will increase the number of patients identified with decreased GFR. Since most ICUs use plasma or serum creatinine for GFR monitoring, they may miss several patients with reduced eGFR_cystatinC_. The discrepancy between the two methods may influence the pharmaceutical treatment of the patients and shows that there is a need to improve GFR measurements in intensive care. Without an optimal GFR marker, using drugs that are less GFR-dependent should be considered. With the existing methodological differences in mind, there is a need for further studies that compare GFR estimated from cystatin C and creatinine with GFR measurement with an exogenous marker such as iohexol, iothalamate, or chromium-51-EDTA in intensive care patients.

## References

[1] du Cheyron D, Bouchet B, Parienti JJ, Ramakers M, Charbonneau P (2005). The attributable mortality of acute renal failure in critically ill patients with liver cirrhosis. Intensive Care Med.

[2] Manhes G, Heng AE, Aublet-Cuvelier B, Gazuy N, Deteix P, Souweine B (2005). Clinical features and outcome of chronic dialysis patients admitted to an intensive care unit. Nephrol Dial Transplant.

[3] Uchino S, Kellum JA, Bellomo R, Doig GS, Morimatsu H, Morgera S (2005). Beginning and Ending Supportive Therapy for the Kidney (BEST Kidney) Investigators. Acute renal failure in critically ill patients: a multinational, multicenter study. JAMA.

[4] Chew DP, Astley C, Molloy D, Vaile J, De Pasquale CG, Aylward P (2006). Morbidity, mortality and economic burden of renal impairment in cardiac intensive care. Intern Med J.

[5] Bäck SE, Krutzen E, Nilsson-Ehle P (1988). Contrast media as markers for glomerular filtration: a pharmacokinetic comparison of four agents. Scand J Clin Lab Invest.

[6] Gaspari F, Perico N, Remuzzi G (1997). Measurement of glomerular filtration rate. Kidney Int.

[7] Toto RD (1995). Conventional measurement of renal function utilizing serum creatinine, creatinine clearance, inulin and para-aminohippuric acid clearance. Curr Opin Nephrol Hypertens.

[8] Cockcroft DW, Gault MH (1976). Prediction of creatinine clearance from serum creatinine. Nephron.

[9] Levey AS, Bosch JP, Lewis JB, Greene T, Rogers N, Roth D (1999). A more accurate method to estimate glomerular filtration rate from serum creatinine: a new prediction equation. Modification of Diet in Renal Disease Study Group. Ann Intern Med.

[10] Hsu CY, Chertow GM, Curhan GC (2002). Methodological issues in studying the epidemiology of mild to moderate chronic renal insufficiency. Kidney Int.

[11] Shemesh O, Golbetz H, Kriss JP, Myers BD (1985). Limitations of creatinine as filtration marker in glomerulopathic patients. Kidney Int.

[12] Sherman DS, Fish DN, Teitelbaum I (2003). Assessing renal function in cirrhotic patients: problems and pitfalls. Am J Kidney Dis.

[13] Grubb AO (2000). Cystatin C-properties and use as diagnostic marker. Adv Clin Chem.

[14] Tenstad O, Roald AB, Grubb A, Aukland K (1996). Renal handling of radiolabelled human cystatin C in the rat. Scand J Clin Lab Invest.

[15] Dharnidharka VR, Kwon C, Stevens G (2002). Serum cystatin C is superior to serum creatinine as a marker of kidney function: a meta-analysis. Am J Kidney Dis.

[16] Villa P, Jimenez M, Soriano MC, Manzanares J, Casasnovas P (2005). Serum cystatin C concentration as a marker of acute renal dysfunction in critically ill patients. Crit Care.

[17] Larsson A, Malm J, Grubb A, Hansson LO (2004). Calculation of glomerular filtration rate expressed in mL/min from plasma cystatin C values in mg/L. Scand J Clin Lab Invest.

[18] Manjunath G, Sarnak MJ, Levey AS (2001). Prediction equations to estimate glomerular filtration rate: an update. Curr Opin Nephrol Hypertens.

[19] Trof RJ, Di Maggio F, Leemreis J, Groeneveld AB (2006). Biomarkers of acute renal injury and renal failure. Shock.

[20] Ahlström A, Tallgren M, Peltonen S, Pettilä V (2004). Evolution and predictive power of serum cystatin C in acute renal failure. Clin Nephrol.

[21] Herget-Rosenthal S, Marggraf G, Hüsing J, Göring F, Pietruck F, Janssen O (2004). Early detection of acute renal failure by serum cystatin C. Kidney Int.

[22] Delanaye P, Lambermont B, Chapelle JP, Gielen J, Gerard P, Rorive G (2004). Plasmatic cystatin C for the estimation of glomerular filtration rate in intensive care units. Intensive Care Med.

[23] Culleton BF, Larson MG, Wilson PW, Evans JC, Parfrey PS, Levy D (1999). Cardiovascular disease and mortality in a community-based cohort with mild renal insufficiency. Kidney Int.

[24] Fried LF, Shlipak MG, Crump C, Bleyer AJ, Gottdiener JS, Kronmal RA (2003). Renal insufficiency as a predictor of cardiovascular outcomes and mortality in elderly individuals. J Am Coll Cardiology.

[25] Jernberg T, Lindahl B, James S, Larsson A, Hansson LO, Wallentin L (2004). Cystatin C: a novel predictor of outcome in suspected or confirmed non-ST-elevation acute coronary syndrome. Circulation.

[26] Larsson A, Helmersson J, Hansson LO, Basu S (2005). Increased serum cystatin C is associated with increased mortality in elderly men. Scand J Clin Lab Invest.

[27] Grubb A, Nyman U, Björk J, Lindström V, Rippe B, Sterner G (2005). Simple cystatin C-based prediction equations for glomerular filtration rate compared with the modification of diet in renal disease prediction equation for adults and the Schwartz and the Counahan-Barratt prediction equations for children. Clin Chem.

[28] Johnson D, Usherwood T (2005). Automated reporting of GFR—coming soon to a laboratory near you! Aust Fam. Physician.

[29] Wulkan R, den Hollander J, Berghout A (2005). Cystatin C: unsuited to use as a marker of kidney function in the intensive care unit. Crit Care.

